# Adjuvant role of a T-type calcium channel blocker, TTA-A2, in lung cancer treatment with paclitaxel

**DOI:** 10.20517/cdr.2021.54

**Published:** 2021-11-14

**Authors:** Neema Kumari, Pravin Shankar Giri, Subha Narayan Rath

**Affiliations:** ^1^Department of Biomedical Engineering, Indian Institute of Technology Hyderabad, Hyderabad 502285, India.; ^2^Department of Biotechnology, Indian Institute of Technology Hyderabad, Hyderabad 502285, India.

**Keywords:** TTA-A2, paclitaxel, adjuvant, lung adenocarcinoma, T-type calcium channel blocker

## Abstract

**Aim:** Chemoresistance is a prevalent issue in cancer treatment. Paclitaxel (PTX) is a microtubule-binding anticancer drug used in various cancer treatments. However, cancer cells often show chemoresistance against PTX with the help of P-glycoprotein (Pgp) - a drug efflux pump. It has also been observed that overexpressed T-type calcium channels (TTCCs) maintain calcium homeostasis in cancer cells, and calcium has a role in chemoresistance. Therefore, the aim of this study was to test the adjuvant role of TTA-A2, a TTCC blocker, in enhancing the anticancer effect of PTX on the A549 lung adenocarcinoma cell line.

**Methods:** Morphology assay, calcium imaging assay, clonogenic assay, apoptosis assay, and real-time polymerase chain reaction (real-time PCR) were performed to find the adjuvant role of TTA-A2. Samples were treated with PTX at 10 nM concentration and TTA-A2 at 50 and 100 nM concentrations. PTX and TTA-A2 were used in the combination treatment at 10 and 100 nM concentrations, respectively.

**Results:** Immunocytochemistry confirmed the expression of TTCC in A549 cells. Morphology assay showed altered morphology of A549 cells. The adjuvant role of TTA-A2 was observed in the calcium imaging assay in spheroids, in the clonogenic assay in monolayers, and in the apoptosis assay in both cultures. With real-time PCR, it was observed that, even though cells express the mRNA of Pgp, it is non-significant upon treatment with PTX and TTA-A2.

**Conclusion:** TTA-A2 can be used as an adjuvant to reduce chemoresistance in cancer cells as well as to enhance the anticancer effect of the standard anticancer drug PTX. Being a potent TTCC inhibitor, TTA-A2 may also enhance the anticancer effects of other anticancer drugs.

## INTRODUCTION

Cancer has a significant impact on mortality worldwide. At least 14 million cancer patients and 8 million cancer deaths are recorded every year^[1]^. Globally, about one in six deaths are due to cancer^[2]^. Chemoresistance to treatment is one of the important factors in increasing cancer-related mortality. Paclitaxel (PTX), a microtubule-binding agent, is widely used in the treatment of various cancers^[3-6]^, such as lung^[7]^, ovarian^[8]^, breast^[9]^, and prostate cancer^[10]^. It binds to the microtubule β-tubulin subunit and stabilizes its structure, thereby preventing microtubule depolymerization. This loss of microtubule dynamics results in the arrest of cells at the G2/M phase of the cell cycle^[7]^. It has also been shown to affect glycolysis through various mechanisms^[11]^. However, cancer cells acquire resistance to PTX through the action of P-glycoprotein (Pgp), a drug efflux pump^[12-14]^. Calcium signaling is another factor known to induce chemoresistance in cells^[14,15]^.

Calcium is an important secondary messenger involved in several cellular processes such as the cell cycle, cell signaling, gene transcription, cell migration, and hormone secretion^[16-18]^. Calcium homeostasis is altered at critical stages of tumor progression and invasion^[18]^. This altered calcium homeostasis helps cancer cells proliferate at a higher rate than normal cells in their microenvironment. It also helps cancer cells to invade other tissues or organs in the body.

The intracellular calcium level, [Ca^2+^]_i_, is maintained by the various calcium channels and pumps present in a cell^[19]^, among which the T-type calcium channel (TTCC) plays an important role in meeting the calcium needs of cancer cells^[20,21]^. TTCC is a low voltage-activated channel that can function at a low resting membrane potential. The role of TTCC in the survival of cancer cells has been established in several studies^[21]^. It has been found that TTCCs are less expressed in differentiated cells (adult stage), whereas they are more expressed in proliferating cells (embryonic stage) and during pathogenesis^[22]^. TTCC is expressed on the cell membrane, through which calcium ions enter cells and further stimulate the release of calcium ions from the sarcoplasmic reticulum, a process termed “calcium-induced-calcium-release”. The released calcium ions then perform cellular functions.

Cancer cells, being highly proliferative, show a 1.5-6.4-fold increase in the expression of TTCCs compared to normal cells^[23]^. There are three isoforms of TTCC: Ca_v_3.1, Ca_v_3.2, and Ca_v_3.3. Different cancer cells have differential expressions of TTCC isoforms^[20]^. Therefore, identifying TTCC isoforms in cancer cells and blocking their function may be an excellent alternative or combination therapy for patients who show resistance to conventional treatment. Drugs that inhibit TTCCs can induce apoptosis and inhibit cell proliferation in some cancers^[21]^.

In recent years, in addition to developing new drugs, studies have also focused on enhancing existing anticancer drugs by combining treatments with other new drugs. Some studies have also shown that using a TTCC blocker sensitizes cancer cells to standard anticancer medication, thus playing an adjuvant role. For example, temozolomide for glioblastoma^[24]^ and carboplatin for ovarian cancer^[25]^ are more effective when given with TTCC blockers.

However, currently available TTCC blockers have insubstantial selectivity for TTCCs. For example, the most popular TTCC blocker, mibefradil, is only 10-20-fold selective towards TTCC compared to high voltage-activated channels. It can also block other channels, such as sodium and potassium channels. Since these sodium and potassium channels are expressed throughout the body, using such blockers can prove fatal. Therefore, in this study, we used TTA-A2, a potent TTCC blocker with ~300-fold affinity for TTCC compared to other channels^[26]^. Our previous study showed its anticancer and adjuvant effects on A549, a lung adenocarcinoma cell line, with the major focus of the study on the killing of cancer cells by TTA-A2^[27]^. In this study, we aimed to show its adjuvant role in enhancing the anticancer property of PTX. This would provide a new treatment regimen in overcoming chemoresistance in cancer cells towards PTX.

## METHODS

### Cell culture and study design

The human lung adenocarcinoma cell line A549 was obtained from the National Centre for Cell Science, Pune, India. The cell line was cultured in Dulbecco’s Modified Essential Medium, high glucose (DMEM-high glucose) (PAN Biotech, Aidenbach, Germany), supplemented with 10% Fetal Bovine Serum (PAN Biotech, Aidenbach, Germany), 100 U/mL Penicillin/0.1 mg/mL Streptomycin (Lonza, Walkersville, MD, USA), and 2.5 mM L-Glutamine (Lonza, Walkersville, MD, USA). The growth conditions were 37 °C, 5% CO_2_, and 100% humidity.

For monolayer culture, 5000 cells/cm^2^ were seeded in the culture dishes. After attachment of cells, dishes were incubated for 24 h before assays. For spheroid culture, 6000 cells/150 μL medium were seeded into 1.5% agarose-coated wells of a 96-well plate and incubated for three days without disturbance before treatment.

PTX and TTA-A2 (Alomone Labs, Jerusalem, Israel) were used as the anticancer drug and the TTCC blocker, respectively. The treatment groups were divided into five groups: Group I, negative control; Group II, PTX 10 nM; Group III, TTA-A2 50 nM; Group IV, TTA-A2 100 nM; and Group V, PTX 10 nM + TTA-A2 100 nM. The drug concentrations used here are based on the IC_50_ values of TTA-A2 obtained in the monolayer and spheroid cultures, as shown in our previous study^[27]^. For the assays, drug stocks were diluted in growth medium to the required concentrations. After the cultures were treated, the drug solutions were replaced with fresh solutions every 24 h. The drug preparation and treatment procedures were carried out in a dark room with minimal light to maintain the effectiveness of the drugs.

### Immunocytochemistry

Non-treated cells cultured in a 96-well plate were with 4% formaldehyde (FA) for 15 min at room temperature (RT). With distilled water, cells were washed for 5 min three times. Triton X-100 (0.1%) was added for 5 min to permeabilize the cell membranes. Cells were washed for 5 min three times with Tris-buffered saline with Tween 20 (TBST). Then, 3% hydrogen peroxide (H_2_O_2_) was added for 20 min. With 2.5% normal horse serum (NHS; Vector Laboratories, Burlingame, CA, USA), cells were kept for 1 h in a humidified chamber. Primary antibodies [Ca_v_3.1, (Thermofisher Scientific, Rockford, USA) and Ca_v_3.2 (Santacruz Biotechnology Inc.)], diluted in a 1:100 ratio in NHS, were added and kept at 4 °C, overnight, in the humidified chamber. Cells were washed for 5 min with TBST three times. Secondary antibodies (Vector Laboratories, Burlingame, CA, USA) were added and incubated for 30 min in a humidified chamber at RT. Cells were then washed for 5 min with TBST three times, and 3,3'Diaminobenzidine (DAB) substrate was added for 2 min. Cells were washed with tap water and counter-stained with hematoxylin for 5 min. Images were taken using a light microscope (Olympus, Tokyo, Japan). The intensity of DAB substrate in each image was measured using Fiji, an open-source platform for biological-image analysis^[28]^.

### Morphology assay

For morphology analysis, images of the monolayer and the spheroid cultures were taken after 48 h of treatment using the light microscope. Monolayer cells were analyzed qualitatively, and the spheroid size was measured using ImageJ software^[29]^.

### Calcium imaging assay

Samples were washed with phosphate-buffered saline (PBS), and 1 μM Fura-2 [acetoxymethyl (AM) ester form] (Life Technologies, Eugene, Oregon), prepared in PBS, was loaded for 1 h at 37 °C in the dark. The dye was replaced with dye-free PBS for 30 min in the dark at RT. Images were taken at the 380/510 and 340/510 nm excitation/emission wavelengths of calcium-free and calcium-bound Fura-2 dye, respectively, using a fluorescence microscope (Carl Zeiss Apotome 2, Germany). The fluorescence intensity was measured using ImageJ software, and the 340/380 ratio was calculated using Equation (1) to determine the relative calcium intensity:

**Figure:**
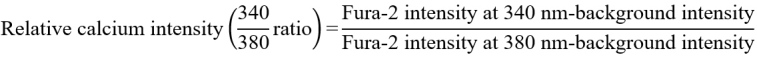


### Clonogenic assay

The protocol given by Sukanya *et al.*^[30]^ was followed. Briefly, cells were seeded in 6-well plates at a density of 250 cells/2 mL medium/well. After 6 h, when cells were attached to the wells, the media was replaced with media from the respective groups. The media was changed every two days. After incubation for eight days, colonies were visualized using Coomassie blue stain. First, colonies were washed with PBS and fixed with 4% FA for 15 min at RT. Coomassie blue stain was added for 20 min, and after washing the wells with deionized water, colonies were counted. Colonies with more than 50 cells were included when counting.

### Apoptosis assay

Monolayer and spheroid cultures were stained using the “Dead Cell Apoptosis kit with Annexin V Alexa Flour™ 488 & Propidium iodide” (Life technologies, Eugene, Oregon) after 48 h of treatment, and fluorescence images were taken. For the monolayer, cells were plated in a 24-well plate at a seeding density of 20,000 cells/well. For spheroid, cells were seeded on 1.5% agarose-coated 96-well plates at a seeding density of 6000 cells/well. After 48 h of treatment, cells were washed with ice cold PBS and stained with Annexin V and PI according to the manufacturer’s protocol. Fluorescence images were acquired using the fluorescence microscope. For analysis, images were converted into 8-bit grayscale and imported into ImageJ. The mean gray value for each channel as well as co-localization was calculated to differentiate between early and late apoptotic cells.

### Real-time PCR

The sequences of TTCC isoforms, Pgp, and β-actin mRNAs were acquired from the National Centre for Biotechnology Information. Primers (sequences listed in Table 1) were synthesized using Primer-Basic Local Alignment Search Tool online software and ordered from Imperial Life Sciences Pvt. Ltd. (Gurugram, Haryana, India).

**Table 1:** Primers for targets, namely Ca_v_3.1, Ca_v_3.2, Pgp, and β-actin, were designed using the Primer-BLAST tool

**Protein**	**Forward sequence 5’- >3’**	**Reverse sequence 5’- >3’**
Ca_v_3.1	TCCGAAAGATTGTGGACAGC	GCTGATTTCTAGGGCGTTGG
Ca_v_3.2	CCCTCATGACCTTCGGCAAC	ACGGCCAGGGAACACATCTTC
Pgp	GGGCTGGATGGCAGTTTTCC	CCCAAAGTCCTTAGCTCCTCC
β-actin	TGTGGCCGAGGACTTTGATTG	AGTGGGGTGGCTTTTAGGATG

Primer-BLAST: Primer-basic local alignment search tools; Pgp: P-glycoprotein.

Cells were grown in duplicate in 6-well plates, and total RNA was extracted manually using TRIzol reagent (Life Technologies, Carlsbad, CA, USA). Next, 1 μg RNA from each group was used to prepare cDNA using the Reverse Transcriptase kit (Qiagen, Hilden, Germany). Real-time PCR was performed using SYBR Green Master Mix (Bio-Rad Laboratories Inc., Hercules, CA) in a real-time PCR instrument (Bio-Rad Laboratories Inc., Hercules, CA). Thermal cycling conditions were as follows: initial denaturation at 95 °C for 2 min followed by 40 cycles of denaturation at 94 °C for 10 s, annealing at 55 °C for 30 s, and extension at 68 °C for 1 min/kb. The final extension was at 68 °C for 10 min. The negative control of each gene was included in the reaction mixture by omitting cDNA samples. mRNA expressions were normalized with β-actin expression. The relative expression of each test gene was measured against the Ca_v_3.1 mRNA expression of the negative control group.

### Statistics

Data are presented as mean ± standard deviation. Unpaired *t*-test was performed for calcium imaging assay, clonogenic assay, and apoptosis assay. One-way analysis of variance was performed for real-time PCR, using OriginPro software^[31]^. A *P*-value < 0.05 was considered significant.

## RESULTS

### TTCC isoforms express differentially in A549 cells

**Figure 1:**
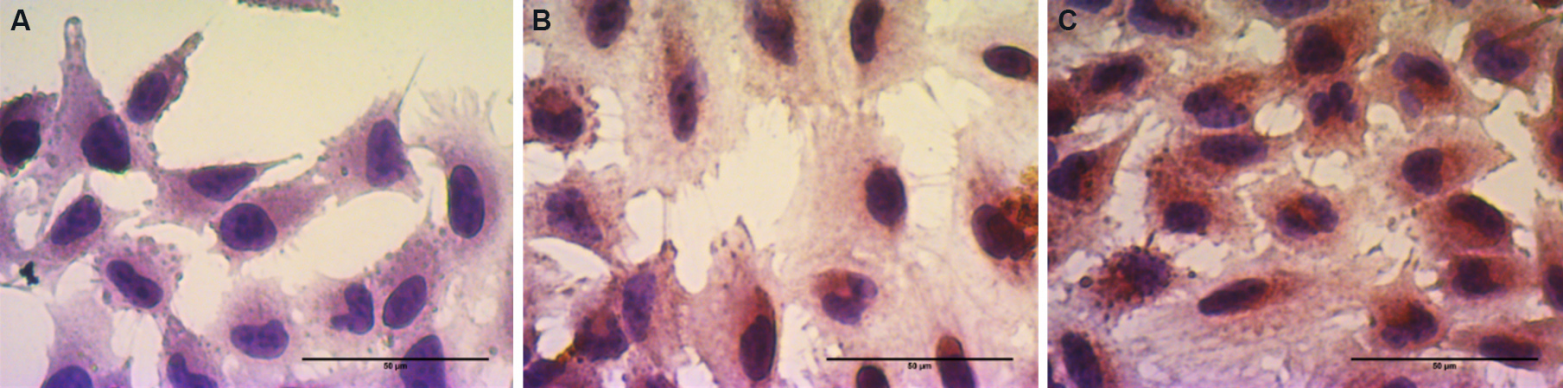
In immunocytochemistry assay, the expression of both Ca_v_3.1 and Ca_v_3.2 was observed in the cells. The no primary antiboday control (A) showed only hematoxylin staining (DAB intensity: 0.273 OD). Ca_v_3.1 antibody-stained cells (B) showed an OD value of 0.349, while Ca_v_3.2 antibody-stained cells (C) showed an OD value of 0.412. Scale bar: 50 μm. OD: Optical density; DAB: 3,3'Diaminobenzidine.

Both Ca_v_3.1 and Ca_v_3.2 isoforms are expressed in A549 cells, as shown in Figure 1. The nuclei of all cells were stained purple with hematoxylin, and DAB coloring was observed in cells stained for TTCC expression [Figure 1B and C]. As measured by Fiji software, DAB intensity (optical density) was 0.273 for no primary antibody control, 0.349 for Ca_v_3.1 antibody-treated cells, and 0.412 for Ca_v_3.2 antibody-treated cells.

Higher intensity of DAB staining with Ca_v_3.2 antibody indicates higher expression of the Ca_v_3.2 isoform compared to the Ca_v_3.1 isoform. The no primary antibody control showed only hematoxylin staining.

### TTA-A2 treatment alters the morphology of monolayer cells

**Figure 2:**
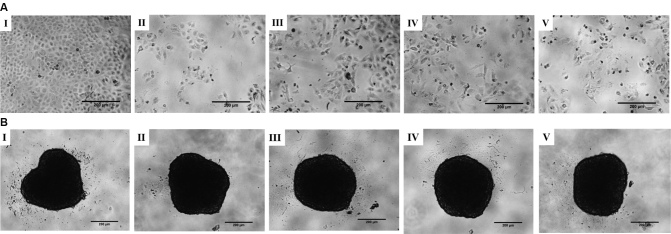
(A) In the monolayer culture, a change in cell morphology was observed in the treatment groups (Groups II-V). Treated cells showed decreased cell density and changed morphology from epithelial form to an elongated or circular shape (solid black cells). (B) In the spheroid culture, all the spheroids had a comparable size of ~300 μm, indicating no treatment effect on reducing the spheroid size, regardless of treatment. Scale bar: 200 μm. I: negative control; II: PTX 10 nM; III: TTA-A2 50 nM; IV: TTA-A2 100 nM; V: PTX 10 nM + TTA-A2 100 nM. PTX: Paclitaxel.

As shown in Figure 2A, in the monolayer culture, the number of cells in each treatment group (Groups II-V) was comparatively less than that of the negative control (Group I). In addition, several cells were rounded in morphology in the treatment groups (Groups II-V). In the case of the spheroid culture [Figure 2B], the size of the spheroids of each group was uniform with a diameter of ~300 μm, indicating no effect on the size of the spheroids by the treatments.

### Combination treatment reduces the relative calcium intensity in spheroids

**Figure 3:**
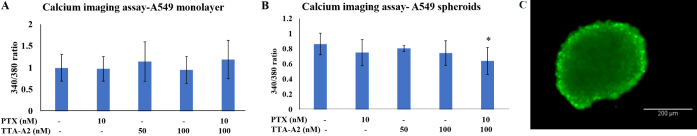
(A) The monolayer culture did not have significantly different 340/380 ratios among the groups. (B) In the spheroid culture, the combination treatment group showed a significantly reduced 340/380 ratio compared to the negative control, where 380 nm indicates the intensity of the Fura-2 dye when not bound to calcium and 340 nm indicates the intensity of the Fura-2 dye intensity when bound to calcium. (C) The Fura-2 dye presented high intensity at the periphery of the spheroid, which is a proliferating region of a spheroid. At the same time, the dye intensity in the core was low. *P < 0.05. Scale bar: 200 μm. PTX: Paclitaxel.

Lower calcium-bound Fura-2 dye intensity (340 nm) was seen in all groups as compared to calcium-free Fura-2 dye intensity (380 nm). Relative calcium intensity (340/380 ratio) was not significantly different among the groups of monolayer cells [Figure 3A]; however, in spheroids, the combination treatment group (Group V) showed a significant decrease in relative calcium intensity compared to the negative control, as shown in Figure 3B. A high intensity of Fura-2 dye was observed in the proliferating region, i.e., at the periphery of the spheroid [Figure 3C].

### Combination treatment significantly reduces Colony Formation Efficiency

**Figure 4:**
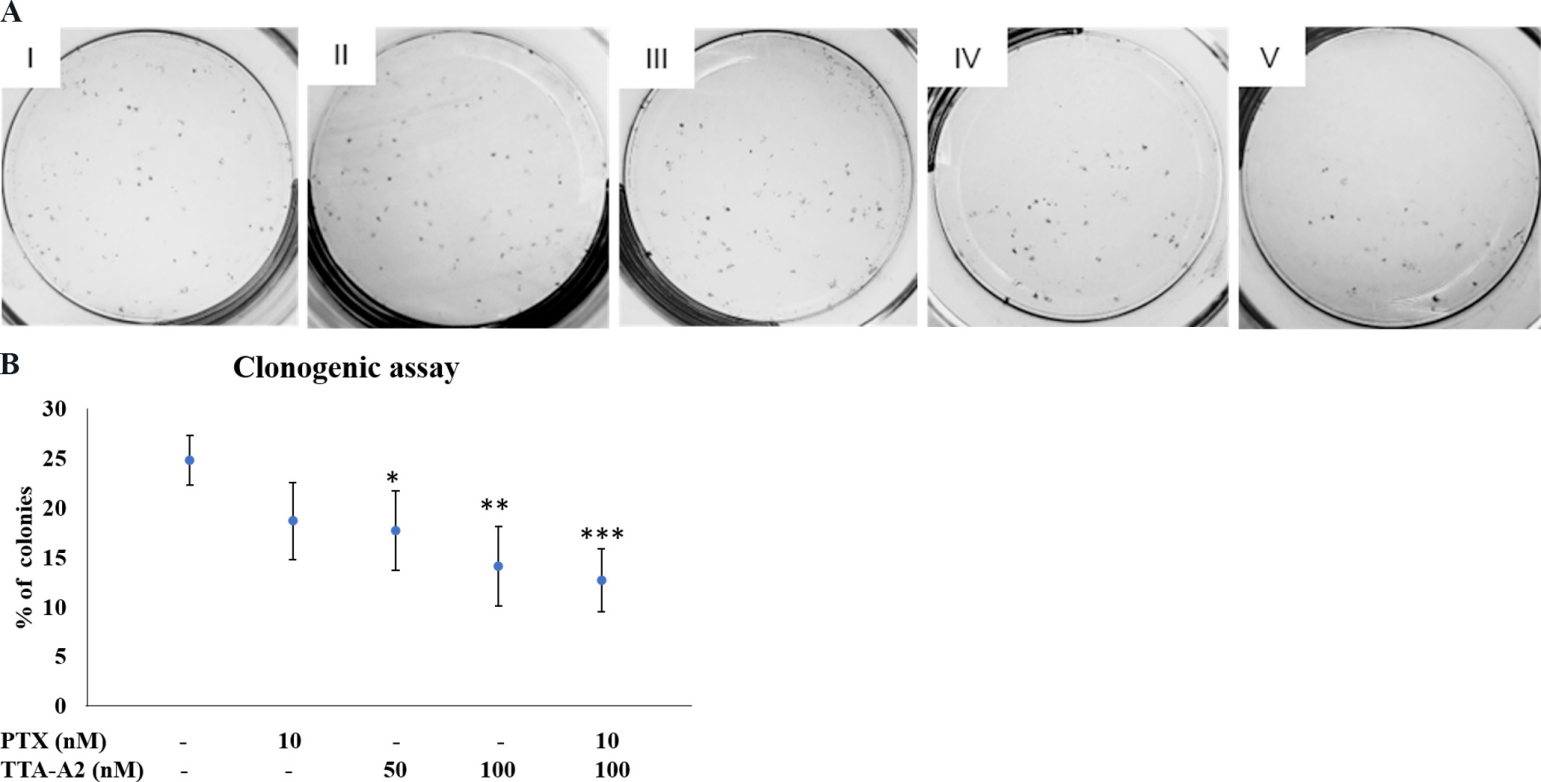
After eight days of the clonogenic assay, colonies were counted (A), and the percent of colonies formed was plotted on a graph (B). Each treatment group reduced the Colony Formation Efficiency of the cells significantly, with the minimum number of colonies in the combination treatment group at 12.7%, compared to the negative control, which had 24.8% of colonies. I: negative control; II: PTX 10 nM; III: TTA-A2 50 nM; IV: TTA-A2 100 nM; V: PTX 10 nM + TTA-A2 100 nM (non-significant *P* > 0.05, *P < 0.05, **P < 0.01, ***P < 0.001). PTX: Paclitaxel.

A significant decrease in the percentage of colonies was observed in the clonogenic assay in all treatment groups, as shown in Figure 4A. The survival of colonies was minimal in the combination treatment group at 12.7%. The percentages of colonies formed in Groups I-IV were 24.8%, 18.7%, 17.7%, and 14.1%, respectively [Figure 4B].

### Combination treatment significantly increases the number of cells in late apoptotic phase

**Figure 5:**
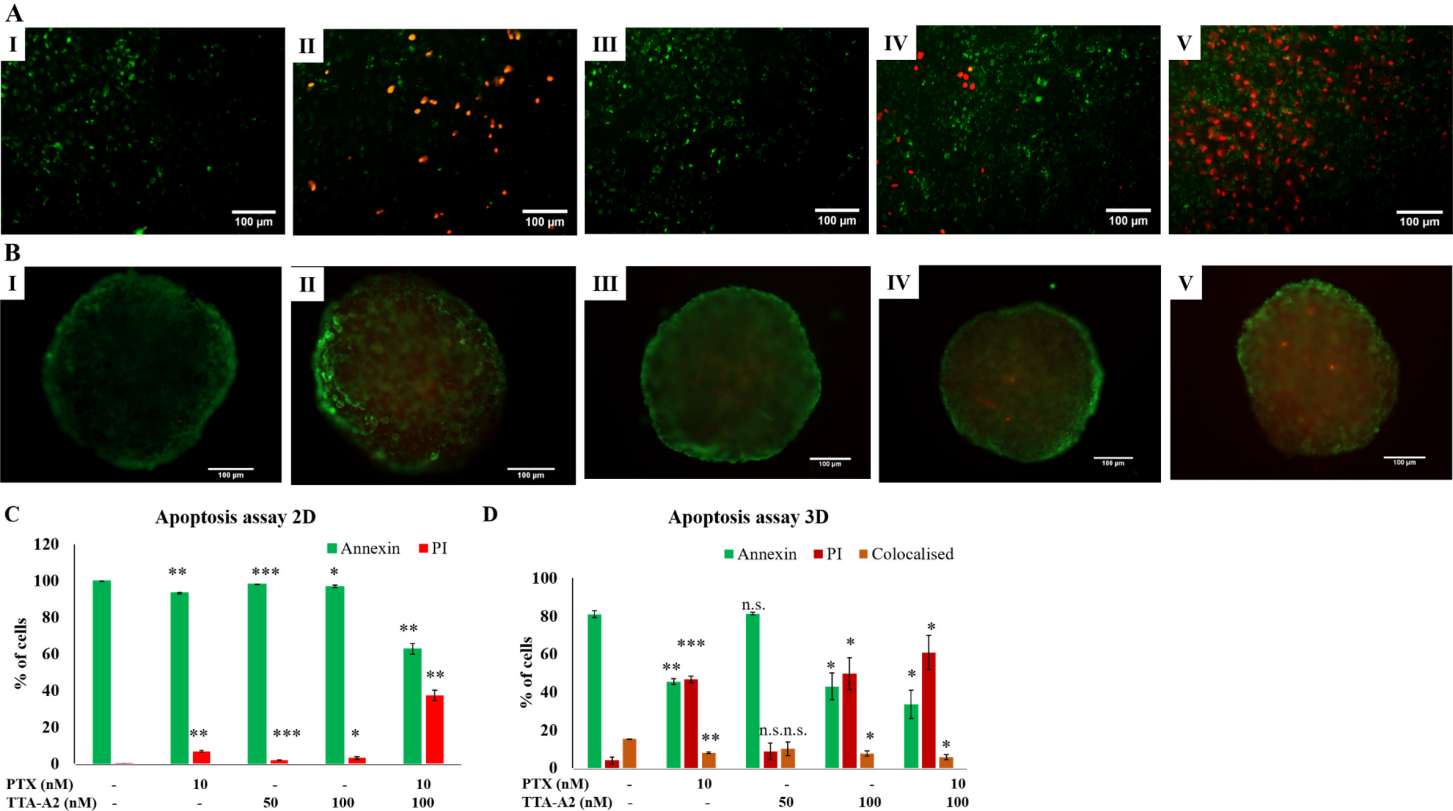
TTA-A2, in combination with PTX, induced increased apoptosis in both monolayer (A, C) and spheroid cultures (B, D) of A549 cells. Cells stained with annexin showed green fluorescence and cells stained with PT showed red fluorescence. In spheroids, several cells showed co-staining with annexin and PI. I: negative control; II: PTX 10 nM; III: TTA-A2 50 nM; IV: TTA-A2 100 nM; V: PTX 10 nM + TTA-A2 100 nM. Scale: 100 μm (non-significant *P* > 0.05, *P < 0.05, **P < 0.01, ***P < 0.001). PTX: Paclitaxel.

As shown in Figure 5, the percentage of cells stained with PI was significantly increased in the combination treatment compared to non-treated cells and other groups in both monolayer and spheroid cultures, indicating that the cells are pushed into late-stage apoptosis upon treatment with TTA-A2 and PTX. In spheroid cultures, co-staining with both dyes was observed in all groups, indicating necrosis in a small percentage of cells.

### TTA-A2 may be effective as an adjuvant without inducing Pgp expression

**Figure 6:**
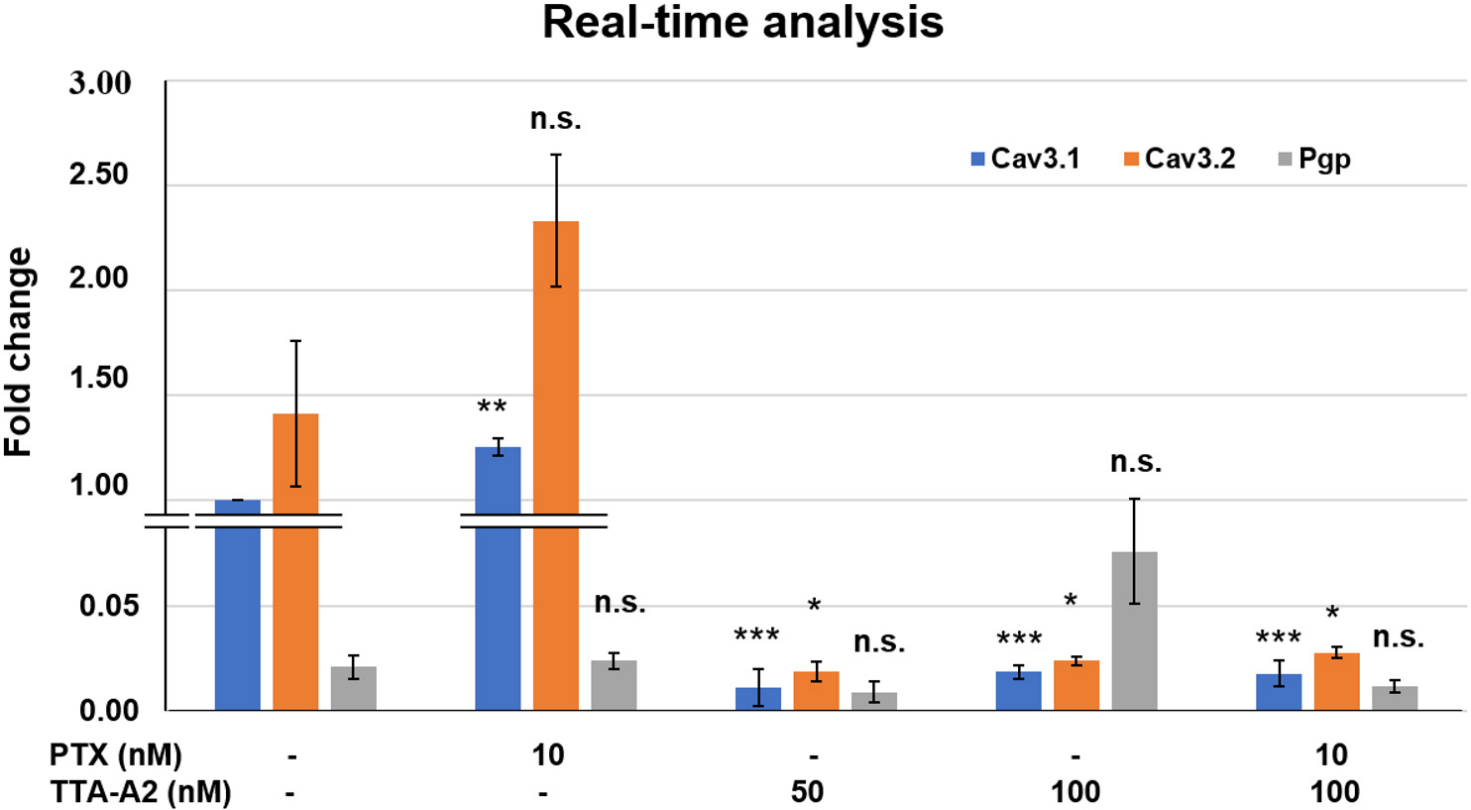
Compared to the negative control, treatment with TTA-A2 significantly reduced Ca_v_3.1 and Ca_v_3.2 mRNA expression in all treatment groups. Furthermore, Pgp expression varied non-significantly among the groups (non-significant *P* > 0.05, *P < 0.05, **P < 0.01, ***P < 0.001). Pgp: P-glycoprotein.

As shown in Figure 6, treatment with TTA-A2 (both alone and in combination) significantly reduced the mRNA expressions of Ca_v_3.1 and Ca_v_3.2. Whereas PTX treatment did not affect the expression of Ca_v_3.2 mRNA, Ca_v_3.1 RNA expression was significantly increased. Pgp mRNA expression was not significantly different among the groups.

## DISCUSSION

The complex nature of cancer poses a great challenge to medical science. The presence of only a few successful anticancer drugs, mutations in genes, and increasing resistance to current chemotherapies make cancer difficult to treat. Therefore, despite massive investments and funds in cancer research and intensive studies on the biochemical, genetic, and functional alterations cancer cells, recovery from cancer is still a major obstacle. In our previous study^[27]^, we showed the anticancer and adjuvant roles of TTA-A2 in lung adenocarcinoma treatment, with a primary focus on the ability of TTA-A2 to induce the cell death of cancer cells. Here, we focused on the adjuvant role of TTA-A2 in enhancing the anticancer effects of PTX.

The edverse effects of treatment can often cause morphological changes in cells or spheroids. When the morphology assay was performed in monolayers, individual treatments changed several cells’ morphology from epithelial to round forms, which visualized these changes. In our previous study^[27]^, we showed through hematoxylin and eosin staining that cells treated with individual drug treatments (Groups II-IV) showed morphology similar to the negative control. However, they did have increased stress-related cytosolic granules and intact cell membrane, and the combination treatment, Group V, showed altered morphology with increased cytosolic granules and blebbing on cell membrane. Here, in the case of spheroids, there was no difference in the size of each group. However, our previous study showed increased intercellular spaces in all the treatment groups (Groups II-V), although there was no difference between treatment groups in terms of increased intercellular spaces. In the same previous study, we also showed in the live/dead assay that both the monolayer and the spheroid cultures in the combination treatment had more dead cells than the other groups.

It is known that growing cancer cells enter the bloodstream to colonize other tissues, a process known as metastasis. This invasive growth or migration of cancer cells is a highly regulated process. Invading cells secrete matrix metalloproteases, which disrupt the extracellular matrix and help cells travel to the surrounding environment^[20]^. Since metastasis is responsible for 90% of cancer-related deaths, targeting metastasis is important in cancer treatment. Therefore, when the ability of TTA-A2 and PTX to inhibit invasion by spheroids was determined, it was found that higher inhibition was observed when both drugs were given together^[27]^. Despite the inhibition, some cancer cells can travel to a new site where they can proliferate to form colonies, which further develop to form solid tumors. Therefore, in this study, the ability of TTA-A2 to inhibit colony formation or reduce the replication capacity of cancer cells was tested. Here, significantly reduced colony formation was observed in all the treatment groups with the fewest colonies in the combination treatment (Group V). The low Colony Formation Efficiency in all the treatment groups indicates a good opportunity to inhibit tumor growth when cells migrate to distant tissue during metastasis. It may also help prevent further growth of tumors when cancer is detected early or prevent tumors from developing in other parts of the body.

No significant reduction in the relative calcium intensity was observed in either treatment group in the monolayer culture. This was probably because the Fura-2-AM dye needs to be converted to its active form, Fura-2, inside the cell. This conversion is carried out by the esterase enzyme, which is present in viable cells. Thus, a dead/non-viable cell cannot convert Fura-2-AM to Fura-2. Therefore, a non-viable cell cannot generate the Fura-2 signal. That is why there was no significant difference in either group as the signal generated was from viable cells. Furthermore, since different calcium channels in a cell modulate [Ca^2+^]_i_^[19]^, the reduction of calcium influx by TTCC blockage may be compensated by other calcium channels. These channels can increase cytosolic calcium levels through influx and efflux from the extracellular region and internal calcium stores, respectively. Cells that lose viability will convert a smaller amount of Fura-2-AM to Fura-2 than a viable cell. Since the detection limit of Fura-2 is 1.2 nM^[32]^, any change in [Ca^2+^]_i_ below 1.2 nM cannot be detected by the dye. The possibility of less viable or non-viable cells being washed away during the washing steps cannot be ignored. Comparing the calcium imaging assay results in the spheroid culture with the monolayer culture results, the combination treatment showed a reduction in the relative calcium intensity, which may be due to less viable cells. Since spheroids have cell-cell contact and cell-matrix contact from all directions, they adhere to each other more strongly; therefore, there is a reduced loss of dead or less viable cells. Therefore, the Fura-2 dye intensity was detected in less viable cells present in the spheroids. In addition, a higher intensity of Fura-2 dye was observed on the surface, indicating a higher calcium level in the outer region of the spheroid, which is also a proliferating region. As TTCCs are overexpressed in proliferating cells, the high intensity of Fura-2 dye on the surface fits well with the theory. In the live/dead assay, in our previous study, spheroids were damaged at the surface on the treatment, indicating the action of TTA-A2 on TTCCs in proliferating cells of spheroids in the outer region. The significant reduction in calcium intensity in the combination treatment also explains the reduced invasion by spheroids in the combination treatment for a longer period^[27]^. In contrast, other treatments inhibited invasion for a short period because the calcium levels reduced by individual treatment were not significant enough.

In the apoptosis assay, a significantly higher number of cells were stained red with PI indicating increased apoptosis compared to the negative control. However, the combination treatment showed a higher percentage of PI-stained cells than all groups, indicating a shift of cells towards the late apoptotic stage. In addition, in the spheroid culture, some cells were co-stained with both dyes in all groups, indicating necrotic cells, which are usually present in the core of spheroids. This result shows that TTA-A2 induces apoptosis in cancer cells as well as when used in combination with a standard anti-cancer drug.

In real-time PCR, the reduced mRNA expression of TTCCs in A549 cells with TTA-A2 treatment indicates that TTA-A2 not only blocks TTCCs but also can regulate their mRNA expressions. This decrease in mRNA expression might be attributed to the binding of TTA-A2 with the G-protein coupled receptor (GPCR). It can be speculated that TTA-A2 acts as an agonist for the GPCR, thus activating it and releasing G_α_ and G_βγ_ subunits. Because of the high expression of TTCC isoforms in A549 cells, signals are sent to inhibit mRNA transcription. However, this speculation of TTA-A2 interacting with GPCR as an agonist needs to be verified.

Real-time PCR also showed that Pgp mRNA expression remained non-significantly changed in each group. Although PTX is known to be one of the substrates for Pgp, perhaps at 10 nM concentration, PTX did not induce overexpression of Pgp. Since TTA-A2 is not a substrate of Pgp^[33]^, using PTX at low concentrations in combination with TTA-A2 may reduce chemoresistance, leading to enhanced anticancer effects in cancer cells.

The above results show the adjuvant role of TTA-A2 in enhancing the anticancer effects of PTX when given together. Although we tested the anticancer effects in combination treatment in this study, these drugs can also be given sequentially, which, as shown in another study, further enhanced the anticancer effect. For sequential treatment, TTA-A2 was given for 24 h and then replaced with PTX for 24 h. The resulting anticancer effect was higher than that of combination treatment^[34]^. This result is similar to previous studies where enhanced effects of standard anticancer drugs were observed when given sequentially with a TTCC blocker. During such treatment, TTCC arrests cancer cells in the G1-S phase. When the blocker is removed, cells re-enter the cell division process in a synchronized manner, thus arresting a higher number of cells at specific phases of the cell cycle. Since most standard anticancer treatments are most effective at a particular phase of the cell cycle, for example, pemetrexed in S phase and radiotherapy in G2/M phase, more cells are killed^[35]^. For some drugs such as temozolomide, prior exposure to a TTCC blocker decreases the time taken to repair DNA damaged by temozolomide^[36]^.

In this study, we found the adjuvant role of TTA-A2 in enhancing the anticancer effects of PTX in the monolayer and the spheroid cultures. Here, homospheroids were used for the study; therefore, further testing TTA-A2 in heterospheorids may better mimic *in vivo* tumors than homospheroids. Cancer stem cells and cancer-associated fibroblasts are also responsible for chemoresistance, invasion, angiogenesis, and cancer relapse^[37,38]^. Therefore, testing TTA-A2 on these cells would also lead to a breakthrough in inducing cell death in these cells.

Overall, this study concludes that TTA-A2 can be used as an adjuvant to reduce chemoresistance in cancer cells as well as to enhance the anticancer effect of the standard anticancer drug PTX. Being a potent TTCC inhibitor, TTA-A2 may also enhance the anticancer effects of other anticancer drugs.

## DECLARATIONS

### Availability of data and materials

The data supporting the study are available from the corresponding author on request. The data are not made publicly available due to privacy or ethical restrictions.

### Copyright

© The Author(s) 2021.
